# Study of PKRBD in HCV genotype 3a infected patients in response to interferon therapy in Pakistani population

**DOI:** 10.1186/1743-422X-10-352

**Published:** 2013-12-09

**Authors:** Atika Mansoor, Lubna Ali, Noor-ul Sabah, Asraf Hussain Hashmi, Mohammad Haroon Khan, Syed Ali Raza Kazmi, Nafees Ahmad, Saima Siddiqi, Khalid Mehmood Khan

**Affiliations:** 1 Institute of Biomedical and Genetic Engineering, 24-Mauve area, G-9/1, Islamabad 44000, Pakistan; 2 Department of Bioinformatics, Muhammad Ali Jinnah University, Islamabad, Pakistan; 3 Pakistan Academy of Sciences, Islamabad, Pakistan

## Abstract

**Background:**

Hepatitis C virus (HCV) is a major cause of liver cirrhosis and hepatocellular carcinoma and infects about 3% world population. Response to interferon therapy depends upon the genotype of the virus and factors associated with the host. Despite a good response to interferon therapy, a considerable number of genotype 3a infected patients remains unalleviated.

**Results:**

In total forty-nine patients including twenty-five non-responders (non-SVR) and twenty-four responders (SVR) were recruited. Patients were tested for viral status at different intervals and the isolated RNA was sequenced for the NS5A region in both groups. The comparison of PKRBD of HCV between the SVR and non-SVR patients did not confirm any significant difference in the number of mutations. However, when the sequence downstream to the PKRBD of NS5A was compared, two important statistically significant mutations were observed; at positions 2309 (Ala to Ser) and 2326 (Gly to Ala). These mutations were then analysed for tertiary protein structure and important structural changes were observed. Statistically significant difference was also observed when age groups of patients were compared; younger patients showed better response than the older ones.

**Conclusions:**

The region between PKRBD and IRRDR may be important for prediction of response to IFN therapy for genotype 3a. ISDR and PKRBD have not shown any involvement in treatment response. Further functional analyses of these findings can help in understanding the involvement of the NS5A region in interferon treatment of HCV-3a infected patients.

## Background

Hepatitis C is a major health problem infecting about 180 million people worldwide and is a leading cause of chronic liver disease and hepatocellular carcinoma [1]. Prevalence of HCV is highest in Egypt, about 22% followed by Pakistan with 4.7% infected individuals. This high carrier rate of infected individuals is due to many reasons including the injudicious use of injections, reuse of syringes and needles, and through shaving tools [2-4].

Interferon-alpha along with ribavirin is commonly used to treat the infected patients. Response to treatment varies from person to person and is also dependent on the viral genotype. HCV genotype appears to be the main predictor of treatment outcome; genotypes 2 and 3 show better response than genotype 1. In Pakistan genotype 3 is the most common type and shows 70-80% response to interferon therapy [5,6]. In spite of a better response compared to other genotypes, a considerable number of individuals remains unrelieved.

Various viral and host factors play a major role in response to interferon therapy; Human leukocyte antigen (HLA) and interleukin 28B (IL28B) being the most commonly studied host factors [7], whereas the viral factors that are frequently studied to predict response to interferon therapy include sequence variability in envelope protein E2, V3 and NS5A regions. Protein kinase R binding domain (PKRBD) of NS5A is a 63 amino acid sequence in which interferon sensitivity determining region (ISDR) is comprised of first 40 amino acids. This domain has been reported to be involved in interaction with protein kinase R (PKR) that inhibits dimerization of PKR and stops its antiviral activity [8-11].

ISDR of the NS5A gene has shown association with non-response in Japanese genotype 1 positive patients [11]. In these patients, the presence of wild type ISDR sequence that is a prototype HCV strain (HCV-J) is an indication of non-response to treatment. However, if there are more than four mutations in the wild type ISDR sequence, there is a better chance of sustained virological response [11]. Few studies carried out to analyse changes in ISDR of HCV-3a showing SVR have presented variable results regarding the involvement of this region in HCV replication and clearance [12-16].

In Pakistan about 10 million individuals are reported to be infected with HCV and the data available on various viral genome factors is not sufficient to determine the response to interferon therapy [2,8]. The present study was planned to investigate the role of PKRBD diversity in response to interferon therapy. In addition, the 3′ sequence outside the PKRBD of NS5A was also analysed to compare the SVR and non-SVR patients infected with 3a genotype.

## Results

### Patients’ response to treatment

Among patients the male and female representation was equal as shown in Table 1. Mean age of non-SVR group was 51 years (range 34–63 years), higher than the SVR group (mean age 43 years, range 21–62). The two groups were compared and statistically significant difference was observed in ages on HCV clearance (p = 0. 003). However, there was no difference in ALT levels (p = 0. 66) at the start of treatment on HCV clearance. Statistically significant differences between SVR and non-SVR patients were also observed in the viral load and rapid virological response (RVR; Table 1). Patients with low viral load and with RVR showed a better response to therapy compared to those with high viral load and no RVR group. Viral loads of both patient groups were compared with the number of mutations in the ISDR, PKRBD and downstream region but no association was found in any of the comparisons.

**Table 1:** Characteristics of the subjects included in the study

		**Non-SVR**	**SVR**	**p-value**
Gender	Males	11 (44%)	11 (46%)	ns
	Females	14 (56%)	13 (54%)	ns
Age	Mean (range)	51 (34–63)	43 (21–62)	p =0.003
ALTs	Mean (range)	119 (45–653)	104 (19–728)	ns
RVR	Achieved (%)	22 (92%)	6 (24%)	p < 0.0001
Viral load	Range	50000-3300000	3300-2000000	p = 0.038

ns, not significant.

### Treatment outcome in relation to PKRBD sequence

**Figure 1:**
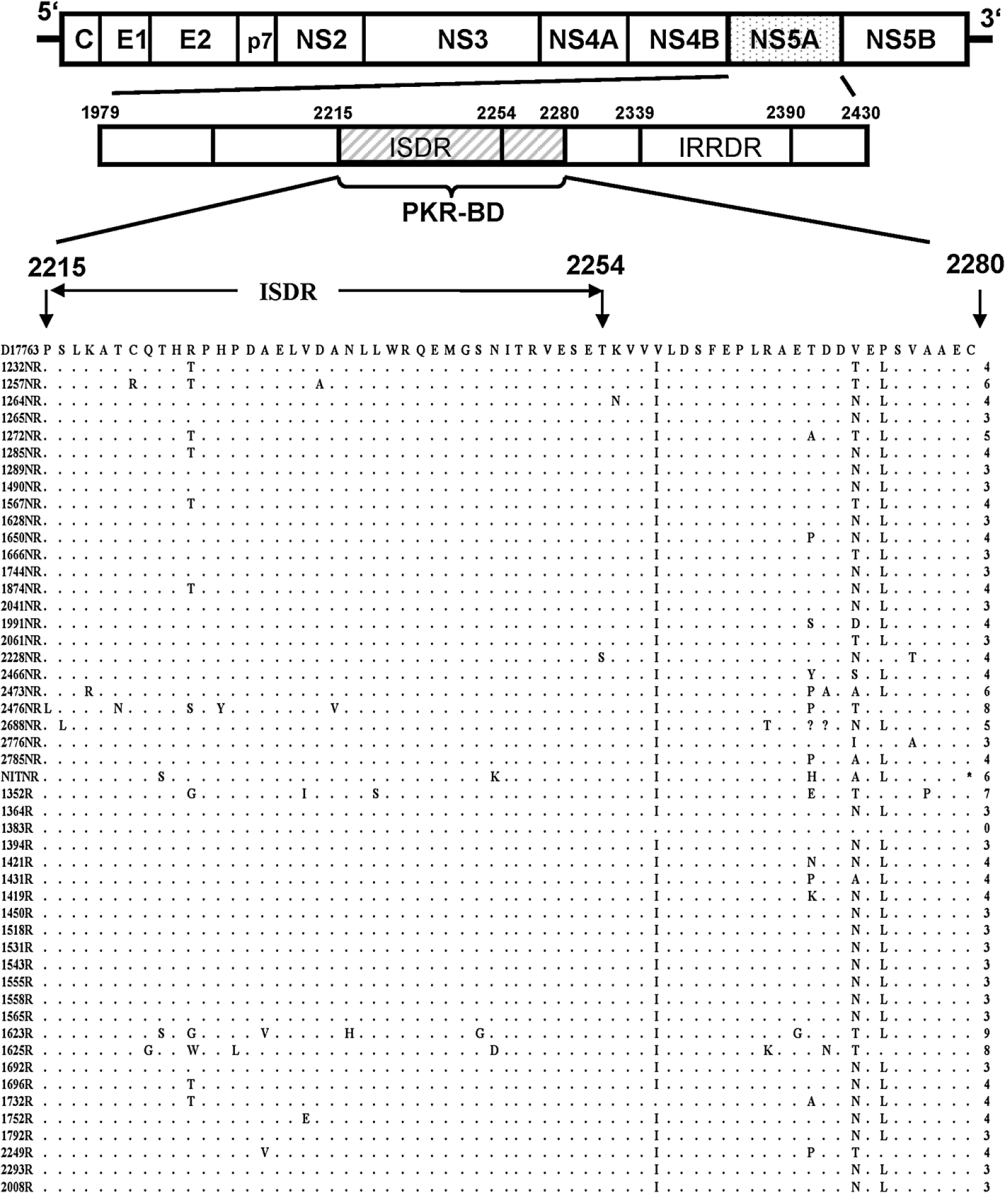
**Sequence alignment of PKRBD sites including ISDR domain.** The SVR and non-SVR are shown as ‘R’ and ‘NR’ in their ID as suffix, respectively. Although there are various mutations in the samples but no statistically significant difference in the number of mutations between SVR and non-SVR patients in ISDR and PKRBD was observed.

**Figure 2:**
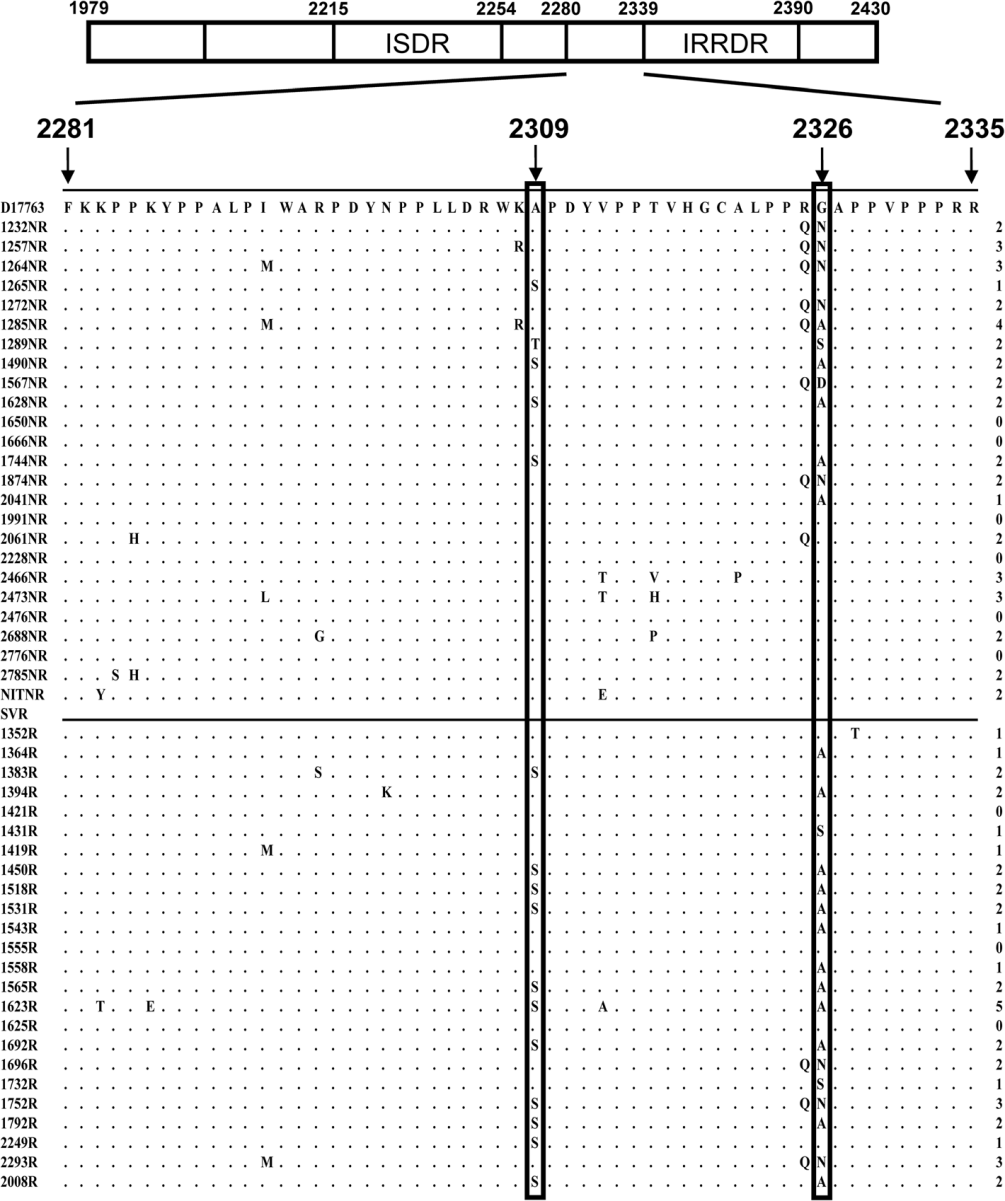
**Comparison of NS5A sequence outside the PKR binding site.** When the NS5A sequence outside the PKRBD (2281–2335) was analyzed, we found the differences between the SVR and non-SVR groups at some amino acid positions. At position 2309 aa Ala to Serine and at position 2326 aa Gly to Ala changes showed statistically significant difference in SVR and non-SVR patients (p ≤ 0.05). Student’s *t*-test was used for statistical analysis.

A 416 bp fragment of the NS5A region covering ISDR and PKRBD was sequenced to find out differences in the number of mutations in SVR and non-SVR patients. These sequences were further divided into different regions; PKRBD, ISDR and region outside PKRBD. Amino acid sequences of the PRKBD were aligned and compared with the already published sequences of the genotype 3a from different regions including India, Brazil, New Zealand and Australia (Additional file 1: Table S1). The sequence identity matrix did not show any significant differences in the amino acid sequence; however, few hyper variable regions were detected in different strains (Additional file 1: Figure S1). The New Zealand strain (NZL1) was almost identical to the already published Pakistani strain (pk1). The NZL1 (GenBank D17763) strain was used for further comparisons and analyses. Few of the mutations specific to Pakistani HCV population are shown in Figure 1. The number of mutations in PKRBD varied from 0–8 in SVR patients (average = 4) and from 0–9 (average = 3.8) in non-SVR patients. There was no statistically significant difference in the number of mutations between SVR and non-SVR patients in ISDR and PKRBD (Figure 1). However, when the sequence outside the PKRBD (2281–2335) was analysed, differences were observed between the two groups at some amino acid positions (Figure 2). There was Ala at position 2309 in the reference strain, whereas a substitution mutation at this point led to a Ser in most of the samples. When this mutation was compared between the SVR and non-SVR patients, significant difference at this point (p = 0.03) was observed. Similarly, at position 2326 Gly was present in reference strain which was replaced by Asn and Ala in many samples. The presence of Ala showed significant association with clearance of hepatitis C virus (p = 0.03). Both of these mutations were higher in SVR samples showing some association with viral clearance in response to interferon therapy.

### Tertiary structure analysis

**Figure 3:**
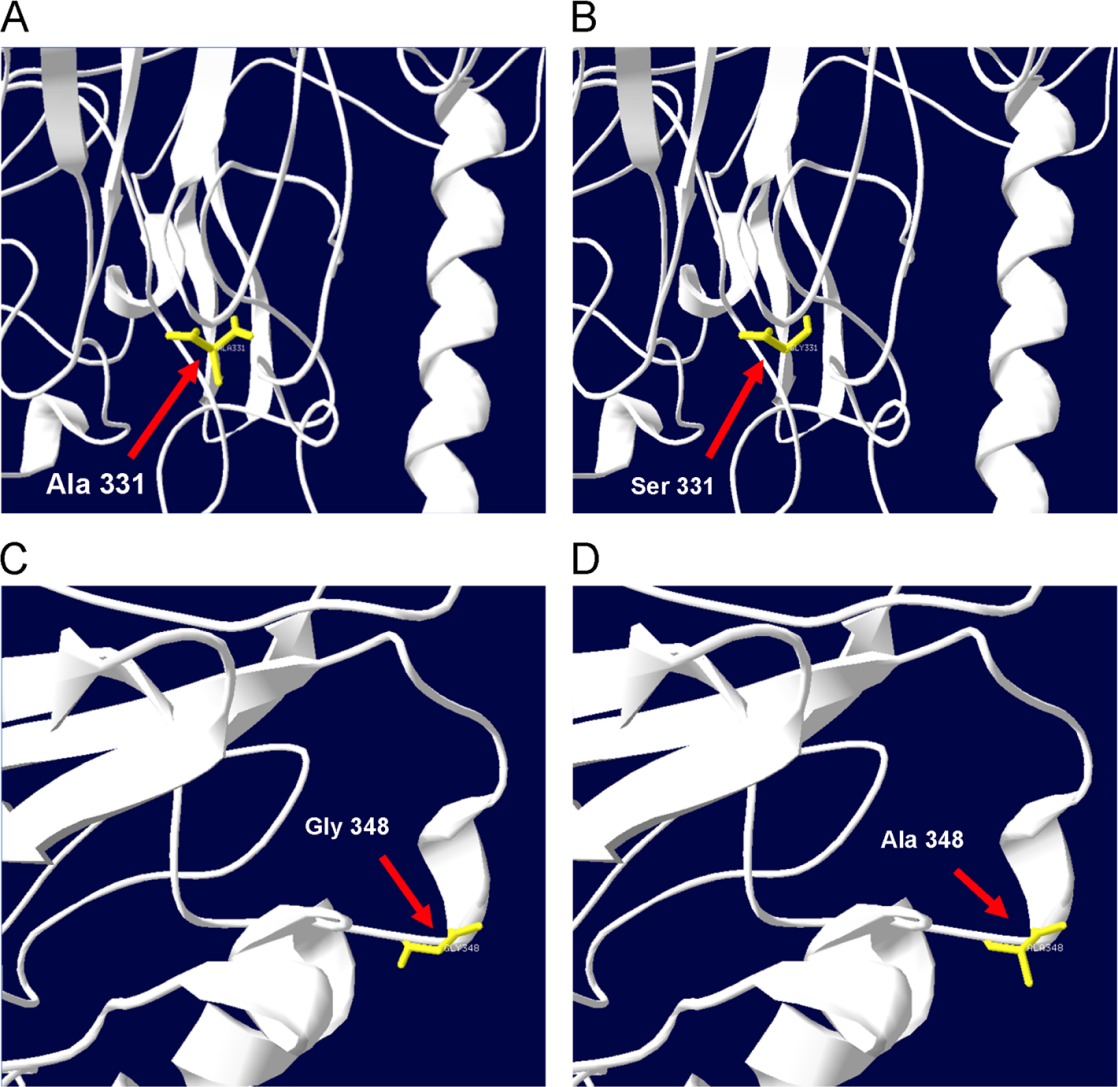
**NS5A structure changes were studied by using mutations present in our samples.** Two of the mutations 2309 (Ala to Ser) and 2326 (Gly to Ala) are present in the transcriptional activation domain. **(A)** Partial structure of NS5A with native residue Ala at position 331 (position 2309 of the poly-protein). **(B)** The first mutation at location 2309 (Ala to Ser) changes the backbone of the amino acid due to the presence of a OH group in the side chain of the new amino acid. The native residue (Ala) is non-polar and more hydrophobic than the mutant, which is a polar one. **(C)** Partial structure of NS5A with native residue Gly at position 348 (position 2326 of the poly-protein). **(D)** Mutation at location 2326 (Gly to Ala). The mutant residue is bigger than the native Glycine. The native residue is the most flexible residue and it is possible that this residue is needed at this position to make a special backbone conformation or to facilitate movement of the protein.

NS5A structural changes were studied by using mutations present in our samples that showed statistically significant association (Figure 3). The A2309S substitution was in a highly conserved region, located within a stretch of residues annotated in Uniprot as a special region: “Transcriptional activation; NS4B-binding”. The mutant residue was bigger and was less hydrophobic than the wild type which might lead to bumps and changes in hydrophobicity. It can also lead to loss of hydrophobic interactions, either in the core or surface of the protein. Similarly, substitution G2326A also generated a bigger residue than the wild type. This substitution was within a stretch of residues annotated in Uniprot as a special region: “Transcriptional activation”. The Gly residue is the most flexible residue and it is possible that this residue is needed at this position to make a special backbone conformation or to facilitate movement of the protein. The mutation introduces a less flexible residue thereby disturbing this conformation or movement of the protein. RMSD value of 0.004 Å was observed for A2309S and 0.002 Å for G2326A. Generally RMSD value between 0 and 1.5 Å represent very similar structures while the increase in RMSD means increased structural dissimilarity. Moreover, small RMSD computed over large structures were also very significant as compared to larger RMSD values computed over structures with a small number of residues. NS5A is a medium sized protein so the observed RMSD values of 0.004 Å and 0.002 Å means non-significant structural variations. The biochemical differences, nature and location of amino acid substitution can affect the protein in various ways and is therefore important to determine whether it can alter the protein function. Theoretical pI of the native NS5A protein was observed as 5.40, which remained unaltered in the mutants, while differences were observed in the alphabetic index and GRAVY. The native protein has an alphabetic index of 66.26 while its GRAVY was −0.418, which were observed deviated in the mutants as 66.04 and −0.424 for A2309S and 66.48 and −0.413 for G2326A, respectively. The alternations of key residues in a protein cause loss of its normal biological functions. It was observed that both the substitutions, A2309S and G2326A were predicted to be TOLERATED with a score of 0.51 and 0.22 by SIFT while regarded as benign by PolyPhen with scores of 0.000 (sensitivity: 1.00 and specificity: 0.00) and 0.001 (sensitivity: 0.99 and specificity: 0.15).

## Discussion

HCV is a major public health problem these days in many countries with global distribution. Generally patients infected with HCV genotype 3a show good response to interferon treatment and the number of non-SVR in HCV 3a patients are quite low; less than 30 percent [5,6]. In order to investigate the non-response of these patients towards interferon therapy, a study was designed in which 25 non-SVR patients with genotype 3a and approximately an equal number of randomly selected SVR patients with the same genotype were included.

Statistically significant differences in the age groups were found when these patients were compared with each other. Response to IFN therapy was greater in younger patients compared to the older ones, as reported previously also [17-19]. It is well known that marked changes in immune response occur with increasing age resulting in blunt immune response. Initial viral load and RVR have been used to predict patients’ final outcome of IFN therapy in many studies and a correlation was also observed in our sample pool [20,21]. Patients with low viral load at the start of the treatment had a greater chance of achieving RVR and subsequent SVR after IFN therapy. Among all the factors analysed RVR was the strongest factor positively associated with viral clearance.

Various studies have been carried out in order to observe the role of ISDR, PKRBD and other sequences present outside the PKRBD of NS5A in response to IFN therapy. Most of these studies have shown association of the number of mutations in these regions to IFN therapy response, especially in the Japanese population and in some European populations as well [11,22-25]. The main aim of the present study was to investigate PKRBD of NS5A sequence that has always been a region of interest due to its involvement in interferon resistance. NS5A is about 447 amino acid long sequence that plays an important role in replication and is divided into three domains separated by two low complexity sequences (LCSI & II; [26]). ISDR and PKR binding site exist between LCSI and domain II [9-11]. ISDR has been previously reported to be involved in treatment response in patients infected with genotype 1 [10]. ElHefnawi *et al*. [27], reported variations in ISDR that were clustered in the SVR groups and some of the positions were associated with viral clearance. One such position significantly associated with subtypes, 1a and 1b was 2228 [27]. When the SVR and non-SVR groups in our sample pool were compared, no difference in the number of mutations in the ISDR region was observed. Other studies on 3a genotype have also shown little or no changes in amino acid sequences when compared with the reference strain (NZL1). Results presented in this study are consistent with previous findings on treatment response of HCV patients to interferon in relation to ISDR sequence variation [12-14]. These results indicate that at least for genotype 3a, ISDR might not be a predictor of SVR in HCV infected patients. PKRBD of NS5A when activated inhibits translation of messenger RNA. Presence of mutations in this region has shown to abolish the interaction between PKR and NS5A. In the present study, the number of mutations in the PKRBD was also not statistically significant suggesting that in our pool PKRBD may not be affected by the number or types of mutations present. Our results are in agreement with other studies showing non-involvement of PKRBD in interferon therapy response [12-14].

We further extended our region of analysis outside the PKRBD and interesting results were found at two positions, which showed statistically significant results. These were substitution mutations, Ala to Ser at position 2309 and Gly to Ala at position 2326. Both of the mutations showed association with viral clearance in SVR patients. Association of mutations outside PKR with treatment outcome have also been reported by Zhou et al., [28] particularly in genotype 1b. El-Shamy et al. [29], have identified a region downstream to ISDR named as IFN/Ribavirin resistance determining region (IRRDR) in genotype 1b. They have further shown variability in this region in genotypes 2a and 2b. IRRDR is extremely variable in most of the HCV genotypes with highly conserved upstream and downstream sequences. The heterogeneity in this region is associated with better treatment response. We have also observed that the mutations identified in the present study are correlated with viral clearance following IFN/RBV treatment regimen (Figure 2), although these mutations are present upstream of the IRRDR region. Therefore, we suggest that the region upstream to IRRDR may also be important in relation to viral clearance with IFN/RBV at least for genotype 3a.

The mutations identified in the sequence were further analysed for their effect on the structure of the protein. Both mutations seemed to affect protein properties. Residue substitutions due to missense mutations can affect the protein high order structure which determines protein functions. HCV NS5A structural variations were therefore analysed by using missense mutations present in our samples that showed statistically significant association (Figure 3). On the basis of these results we can only predict that the presence of these mutations is somehow affecting the interferon effect in HCV infected patients. These mutations are probably giving selective protection to the patients who in turn show better response towards interferon therapy. On the other hand absence of these mutations in NS5A leads to non-response. Besides the viral factors, host factors including HLA alleles and interleukins (especially IL28B) can also be helpful in predicting the IFN therapy outcome. Polymorphisms in and around IL28B gene have shown an association to IFN therapy; rs12979860 being most widely studied [30-32]. However, no data are available for polymorphisms in IL28B from Pakistan so far. Therefore, study of these polymorphisms will be helpful in predicting the treatment response in HCV patients and these viral and host factors together can help in deciding the treatment regimen for the patients of this region.

## Conclusion

In conclusion, we can say that ISDR and PKRBD of HCV-3a genotype are not predictive markers for the outcome of interferon therapy. However, the downstream region to the PKRBD of NS5A might be playing some role in this regard. The mutations in the downstream region of NS5A presented here and their relation to interferon therapy are reported for the first time. Further studies are needed to confirm these results and an *in vitro* cellular model system is needed to verify the effect of these mutations.

## Methods

### Subjects and response classification

A total of 49 HCV patients infected with HCV (3a) genotype were included in this study. Blood samples of the patients were collected after the patients gave their written informed consent. This study was approved by the Institute of Biomedical and Genetic Engineering Ethical Committee and was in compliance with the Helsinki Declaration. All patients included in the study were treatment naïve and were HCV 3agenotypepositive. In Pakistan it is a routine practice in most of the hospitals to give standard interferon therapy that includes three million unit standard IFN three times a week with 600 mg ribavirin twice a day for at least six months (72 injections). All of the patients were tested for HCV RNA at the start of the treatment, after four weeks for a rapid virological response (RVR) and then at the end of therapy for end of treatment response (ETR). At the end of treatment the patients who did not clear the virus were treated as non-sustained virological response (non-SVR). Whereas, the patients who responded to the therapy were monitored for sustained virological response (SVR) six months after the end of treatment and were grouped as responders. Samples for the study were collected during the year 2008 to 2011 and the viral load of the patients was determined by Qiagen kit (Artus HCV RG RT-PCR kit). The exact route of viral infection is not clear; however, most of the patients had the history of dental treatment or surgery. Basic clinical parameters of the patients are given in Table 1.

### HCV detection and genotyping

Plasma/serum from HCV infected patients were separated within 1 hour of blood collection and stored at −20°C. HCV RNA was isolated using a commercially available QIAamp Viral RNA Mini Kit (Qiagen Cat. # 52906) and detected by Qiagen kit (Artus HCV RG RT-PCR kit). HCV genotyping was performed as described previously [33]. This method detects genotypes 1a, 1b, 2a, 2b, 3a, 3b, 4, 5a and 6.

### Primer designing for NS5A

D17763 NZL sequence of HCV-3a was used as a reference. NS5A sequence including ISDR region was retrieved from NCBI database and primers were designed using primer 3 software available online [34]. Primers selected for amplification of about 416 bp were as follows: forward primer (5′ CGCGGGTCMCCTCCATCAGA 3′) and reverse primer (5′ TTCCTCCGRGGGGAGGCAC 3′). These primers were synthesized from MWG operon (Biotech, UK).

### PCR amplification of NS5A region and sequencing

Purified RNA samples were subjected to reverse transcription and amplification by using a OneStep RT PCR kit from Qiagen (Cat #210212). Briefly RNA samples were reverse transcribed at 50°C for 30 minutes, denatured at 95°C for 15 minutes and amplified for 40 cycles, each consisting of denaturation at 94°C, annealing at 60°C and extension at 72°C for 1 minute each. Amplified products were confirmed on 2% agarose gel and were then sequenced bidirectionally using standard protocols. Sequenced products were separated on 3130 Genetic Analyzer (ABI part no. 4363785), data were collected and analysed by ABI genetic analysis software. The sequences obtained were compared manually to the reference sequence D17763 NZL.

### Statistical analysis

Amino acid sequences of samples were aligned by Molecular Evolutionary Genetics Analysis software (MEGA version 5 [35]) and aligned sequences were exported to Microsoft excel for calculation of number of mutations and their averages. For comparison between responders and non-responder groups, Fisher exact test was applied using VassarStats (http://faculty.vassar.edu/lowry/tabs.html) and P ≤ 0. 05 was considered as statistically significant. Two tailed Student’s *t*-test was used to assess differences in age groups and ALT levels.

### Sequence and structural analysis

Sequence of HCV NS5A protein with accession number D17763 was retrieved from UniProt (http://www.uniprot.org) database for detailed structure based assessment. For a detail insight, three dimensional structure of HCV NS5A protein was predicted through I-TASSER server [36], refined through ModRefiner [37] for quality enhancement and validated for quality assurance through WHATIF [38].

The observed mutations, A2309S and G2326A were substituted in the native sequence of HCV NS5A using MUTATE_MODEL [39] to get the mutant versions for investigating structural and functional deviations. The mutant models were compared against the native in 3D through PDBeFOLD (http://www.ebi.ac.uk/msd-srv/ssm) for structural similarities. Physiochemical properties were predicted through ProtParam (http://au.expasy.org/tools/protparam.html). Sorting intolerant from tolerant amino acid substitutions based on sequence homology was predicted through SIFT [40] to predict whether an amino acid substitution in a protein will have a phenotypic effect. The damaging substitutions were further sorted from the benign by using PolyPhen server (genetics.bwh.harvard.edu/pph/), which predicts the possible impact of residue substitution on the structure and function of a human protein.

## Competing interests

Authors have no competing interests.

## Authors’ contributions

AM and KMK designed the study, analysed the data and drafted the manuscript. LA, NS and AHH co-ordinated the laboratory experiments. MHK, NA, SS helped in statistical and structural analysis. ARK organized sample collection from the hospital. All authors read and approved the final manuscript.

## Supplementary Material

Additional file 1 **Supplementary material.**http://www.virologyj.com/imedia/1948169713111749/supp1.doc. Click here for file
