# Supplementary material for: Study of PKRBD in HCV genotype 3a infected patients in response to interferon therapy in Pakistani population
Source: Virol J. 2013 Dec 9;10:352. doi: 10.1186/1743-422X-10-352 (PMC4029318; doi:10.1186/1743-422X-10-352)
Supplement: Additional file 1 — Supplementary material.http://www.virologyj.com/imedia/1948169713111749/supp1.doc. [file 1743-422X-10-352-S1.doc]

**Supplementary material**

**Study of PKRBD in HCV genotype 3a infected patients in response to Interferon therapy in Pakistani population**

Atika Mansoor^1§^, Lubna Ali^1^, Noor-ul-Sabah^1^, Asraf Hashmi^1^, Mohammad Haroon Khan^2^, Syed Ali Raza Kazmi^1^, Nafees Ahmad^1^, Saima Siddiqi^1^ and Khalid M. Khan^3^

^1^Institute of Biomedical and Genetic Engineering, Islamabad, Pakistan, ^2^Department of Bioinformatics, Muhammad Ali Jinnah University, Islamabad, Pakistan, ^3^Pakistan Academy of Sciences, Islamabad, Pakistan

**Table S1:** Sequence identity matrix of ISDR of NS5A of different strains of HCV-3a genotype.

| **Sequence** | **IBGE-PK-HCV2008R** | **pk1** | **NZL1** | **Indian 3a** | **Brazil-RF145C26** | **Australian3a strain CB** | **K3a** |
| --- | --- | --- | --- | --- | --- | --- | --- |
| **IBGE-PK-HCV2008R** | ID | 0.958 | 0.958 | 0.975 | 0.966 | 0.966 | 0.966 |
| **pk1** | 0.958 | ID | 1.000 | 0.975 | 0.966 | 0.966 | 0.950 |
| **NZL1** | 0.958 | 1.000 | ID | 0.975 | 0.966 | 0.966 | 0.950 |
| **Indian 3a** | 0.975 | 0.975 | 0.975 | ID | 0.983 | 0.991 | 0.975 |
| **Brazil-RF145C26** | 0.966 | 0.966 | 0.966 | 0.983 | ID | 0.975 | 0.958 |
| **Australian-3a strain CB** | 0.966 | 0.966 | 0.966 | 0.991 | 0.975 | ID | 0.966 |
| **K3a** | 0.966 | 0.950 | 0.950 | 0.975 | 0.958 | 0.966 | ID |

**Figure S1: Alignment of ISDR of NS5a of different strains of 3a genotype**. No gross difference in different stains was observed. However, two variable regions were observed at position 2272 and 2326.
